# Beyond Single-Platform Adoption: Unpacking Doctors’ Cross-Channel Behavioral Mechanisms in Omni-Channel Medical Practice

**DOI:** 10.3390/healthcare14070923

**Published:** 2026-04-01

**Authors:** Jianmei Du, Shuwan Zhu

**Affiliations:** School of Management, Hefei University of Technology, No. 193 Tunxi Road, Hefei 230009, China; zhushuwan@hfut.edu.cn

**Keywords:** Omni-Channel Online Medical (OCOM) service mode, UTAUT, channel effect, technology transfer, structural equation model

## Abstract

**Highlights:**

**What are the main findings?**

- Doctors’ adoption of the OCOM service mode is mainly driven by effort expectancy, social influence, habit, and patient volume.
- Performance expectancy is no longer a significant factor in the pandemic context.
- Social platform use promotes doctors’ engagement with professional platforms.

**What are the implication of the main findings?**

- Platforms should prioritize ease of use and reduce operational complexity.
- Enhancing social influence and cross-platform integration can promote OCOM adoption.

**Abstract:**

**Background:** As digital healthcare ecosystems evolve, doctors are no longer confined to a single platform for online medical services. Instead, a new omni-channel service mode has emerged, integrating professional medical platforms with widely used social platforms. This shift raises important questions about how doctors adopt and navigate multi-platform environments. The purpose of this study is to explore the behavioral mechanisms that shape doctors’ adoption of omni-channel online medical services in the post-pandemic era. **Methods:** Drawing on the Unified Theory of Acceptance and Use of Technology, complemented by channel effect and technology transfer perspectives, we conducted a survey of 958 Chinese doctors. A structural equation model was employed to test the relationships among effort expectancy, social influence, patient volume, habitual use, platform experience, future expectancy, and adoption intention. **Results:** The analysis revealed that effort expectancy, social influence, patient volume, and habitual use exert significant positive effects on adoption intention. Platform experience enhances doctors’ perceptions of ease of use and usefulness, while future expectancy indirectly shapes adoption intention through these perceptions. In contrast, performance expectancy no longer emerges as a decisive factor in the post-pandemic context, suggesting that external motivations may be overshadowed by practical experience and social dynamics. Moreover, doctors’ engagement with social platforms positively influences their use of professional platforms, highlighting cross-channel spillover effects that reinforce adoption across service types. **Conclusions:** This study extends technology adoption theory by situating doctors within dynamic, multi-platform service environments and demonstrating the importance of cross-channel influences. The findings provide practical guidance for platform designers and policymakers on how to effectively integrate professional and social platforms to enhance digital healthcare delivery.

## 1. Introduction

**Figure 1:**
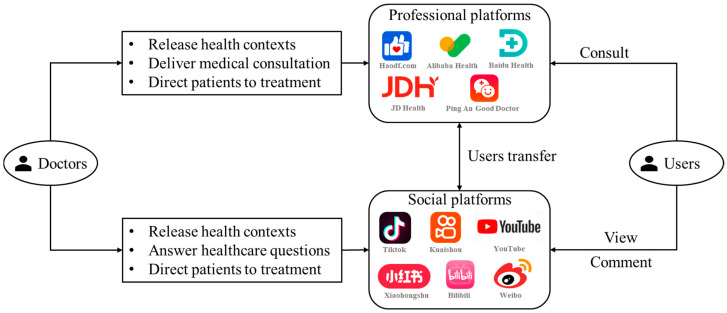
Illustration of the OCOM service mode.

With the widespread application of emerging digital technologies in the healthcare domain, such as artificial intelligence, blockchain, and data-driven systems [1,2,3], the digital transformation of healthcare has redefined how medical services are delivered, with online platforms playing an increasingly pivotal role, especially during the pandemic [4]. More people are now turning to online channels to access health information, receive diagnoses, and seek medical treatment [5]. These services are no longer limited to professional medical platforms, such as Haodaifu Online and Ping’an Good Doctor in China, or Teladoc Health and ZocDoc in the USA [6]. Increasingly, social media platforms, including Sina Weibo, Douyin, Xiaohongshu, YouTube, Twitter, and Facebook, are being used to access, share, and discuss medical information [7,8]. This growing integration of diverse digital platforms into healthcare has fostered a widespread habit of seeking medical support online, prompting the emergence of the Omni-Channel Online Medical (OCOM) service mode, in which doctors strategically utilize multiple online channels to extend their reach, build trust, and communicate more effectively with patients beyond institutional boundaries. The COVID-19 pandemic further emphasizes the necessity for doctors to provide seamless and personalized healthcare services through diverse online platforms [9]. Figure 1 illustrates the structure of the OCOM service mode.

Unlike traditional single-channel services, the OCOM service mode integrates multiple online ecosystems, enabling doctors to reach a broader patient base, extend their professional influence, and diversify their revenue streams [8,10,11]. Such a service mode has been gradually employed to serve various purposes, including increasing sales, ensuring consumer satisfaction and loyalty, improving operational effectiveness [12], and enhancing efficiency in resource utilization [13]. However, this cross-platform integration also introduces new complexities, as different platforms vary significantly in terms of technical features, user demographics, and interaction norms. These differences pose strategic and operational challenges for doctors navigating this multi-platform landscape. Therefore, understanding how medical professionals adopt and manage OCOM practices across diverse platforms is crucial for advancing the effectiveness and sustainability of digital healthcare systems.

However, the adoption of the OCOM mode is far from uniform. While some doctors embrace social platforms for their broader reach and patient traffic, others remain loyal to professional platforms valued for their medical credibility and regulatory assurance [8,14]. This divergence reflects the complex strategic calculus that doctors must navigate when operating in multiple digital environments. The tension between platform professionalism and audience scale underscores the need to understand what drives doctors’ platform preferences. The COVID-19 pandemic further intensified this complexity, accelerating OCOM adoption while potentially reshaping service expectations and usage habits.

Despite the growing relevance of the OCOM mode [11], prior research has primarily examined doctors’ use of either professional or social platforms in isolation [15,16], with limited attention to how behaviors and experiences may transfer across platforms [17]. Moreover, the COVID-19 pandemic has not only accelerated the uptake of digital healthcare technologies but also introduced contextual shifts, such as increased time pressure, institutional uncertainty, and changing user expectations, which may affect how doctors evaluate and adopt new digital tools [18]. These dynamics call for a reassessment of conventional technology adoption frameworks in light of multi-platform realities. While professional and social platforms differ in function and audience, they also share common features in medical content creation, consultation tools, and user interaction. These shared affordances enable doctors to transfer experience and skills from one platform to another, reducing switching costs and facilitating cross-platform engagement. Consequently, OCOM adoption involves not only external contextual factors, but also internalized patterns of technology transfer, which cannot be fully captured by conventional single-channel models.

To address these gaps, this study investigates the factors influencing doctors’ adoption of the OCOM service mode by integrating the Unified Theory of Acceptance and Use of Technology (UTAUT) [19] with theories of channel effect [20] and technology transfer [21]. Specifically, we focus on the role of effort expectancy, social influence, habit, platform experience, and patient volume in shaping adoption intentions. We also examine how experience with one type of platform influences usage of the other, capturing interaction effects across channels. By modeling doctors as cross-platform actors in a converging service ecosystem, we contribute to a deeper understanding of digital healthcare adoption in the post-pandemic era.

Building on this framework, our study makes several important contributions to the literature on digital healthcare adoption. Theoretically, we extend the Unified Theory of Acceptance and Use of Technology (UTAUT) by embedding it within a cross-platform behavioral model that incorporates channel effect theory and technology transfer mechanisms, allowing us to capture how doctors’ prior experience with one platform shapes their engagement with another. This integration enriches existing adoption theories by addressing the interactional nature of service behaviors in multi-platform environments. Empirically, we examine how key psychological and contextual variables, such as effort expectancy, social influence, usage habit, and patient volume, affect doctors’ intention to adopt the Omni-Channel Online Medical (OCOM) service mode. Our findings further demonstrate that, in the post-pandemic context, traditional predictors such as performance expectancy have become less salient, while social and experiential factors have gained prominence. Finally, we identify an asymmetric but reinforcing cross-platform pattern: doctors’ engagement with social platforms positively influences their use of professional platforms, revealing a “spill-in” effect [17] that has practical implications for platform design, user migration strategies, and digital health policy.

The remainder of this paper is organized as follows: First, we review the literature and propose hypotheses accordingly. Then, we describe the research methodology and report the analysis results. Finally, we present the key findings, implications, and limitations of our study.

## 2. Literature Review and Research Hypotheses

In this section, we aim to summarize the multi-channel mode in various fields and the theoretical models of information technology, providing a solid theoretical foundation for our research on doctors’ adoption of the OCOM service mode. We then develop our research hypotheses according to these theoretical models.

### 2.1. Omni-Channel Services

Omni-channel service is generally considered to utilize multiple channels in the communication and interaction process between providers and consumers to provide/obtain products or services [11]. Many scholars have focused on the omni-channel service mode. In addition to implementing omni-channel services in retailers [22], this service mode also facilitates online healthcare. The pandemic further emphasizes the need for doctors to adopt an omni-channel service strategy [9]. Ref. [13] explored patients’ acceptance and usage of telemedicine within the context of the omni-channel decision process, which encompasses the use of social media, websites, and internal health channels for medical services. Ref. [23] focused on the effects of integrating online and offline services on the demand and reputational outcomes of e-healthcare providers. Although researchers have studied omni-channel services in the medical and retail sectors, they have mainly focused on integrating online and offline channels, with little attention paid to the impact of integrating online medical channels. To fill this gap, in this paper, we focus on the OCOM service mode, an online service mode for doctors to communicate or post health-related content through various channels, which includes but is not limited to professional platforms and social platforms, and then explore the influence factors and mechanisms on the doctors’ adoption intention of this service mode.

### 2.2. Service Adoption Models

The user is a critical factor in online medical platforms; hence, the willingness of doctors to use the OCOM service mode is closely related to the development of online medical services, which is conducive to alleviating pressure on medical resources in the healthcare field. Several models are employed in research on technology adoption and use, including the Theory of Reasoned Action (TRA), the Theory of Planned Behavior (TPB), and the Technology Acceptance Model (TAM). Specifically, the Unified Theory of User Acceptance of Technology (UTAUT) has been introduced to measure how users adopt and engage with information technology and systems [19]. In this section, we review several of the most popular technology adoption models, such as the TRA, TPB, TAM, and UTAUT.

TRA is proposed by [24] based on social psychology and is widely used to predict consumer adoption intention. It primarily hinges on three core elements: subjective norms, attitudes, and adoption intentions. Subjective norms refer to the influence of social environments, such as friends, peers, and family, on attitudes that explain human adoption intentions, both positive and negative [25]. TPB is an extension of the theory of reasoned action (TRA) in addressing the limitations of TRA regarding human behavior with incomplete over-volitional control [26]. According to the TPB, consumers’ actual control and adoption intentions determine their behaviors, with intentions being influenced by attitude, subjective norm, and perceived behavioral control [27].

TAM was introduced by [28] and is typically used to predict consumers’ behavior when accepting new information technologies, considering that the key to increasing use is to first increase acceptance of the technology [29]. In the original and extended TAM model, perceived usefulness and perceived ease of use are two key factors that determine consumers’ intention to use, which is considered a factor influencing their actual usage behavior, unlike the TRA and TPB models [30]. This model, along with the TPB and other technology adoption models, plays a crucial role in exploring users’ intentions to adopt new health-related information technologies, such as AI-based medical diagnosis support systems [31].

UTAUT is a comprehensive model proposed by [19], which integrates key determinants from eight technology acceptance models (TRA, TPB, TAM, etc.) [32], suggesting that consumers’ adoption intention and their ultimate behavior are directly determined by performance expectancy, effort expectancy, social influence, and facilitating conditions. A considerable body of literature employs this model and its extended frameworks to elucidate the behaviors of users adopting new technologies across diverse domains. Ref. [33] provided a good example of the application of UTAUT in IT acceptance research by keeping some UTAUT constructs, such as performance expectancy, social influence, and facilitating conditions (but not effort expectancy), to adapt to their research context. These adjustments provide a basis for the flexible use of UTAUT in our study.

## 3. Research Model and Hypotheses

In this section, we develop our research model based on various theories, including the UTAUT and the TAM, combined with research context characteristics and previous research. The model encompasses the OCOM service mode adoption factors and hypotheses.

### 3.1. UTAUT and the OCOM Service Mode Adoption

In the UTAUT model, performance expectancy, effort expectancy, and social influence directly influence adoption intention, which influences actual behavior. In contrast, facilitating conditions directly influence actual behavior. Gender, age, experience, and voluntariness of use are posited to moderate these constructs [34]. This model effectively considers individuals’ user environment by explaining their technology adoption using internal, external, and social dimensions [35]. These tenets of the UTAUT model make it applicable for studying the adoption of the OCOM service mode in this study. We control for certain UTAUT constructs, including performance expectancy, effort expectancy, and social influence (but not facilitating conditions), which may impact doctors’ adoption intention toward the OCOM service mode in our context. In addition to exploring the interaction effects among different channels, our research model also focuses on doctors’ actual use of the OCOM service mode across two types of online channels. In this paper, adoption intention refers to doctors’ willingness to adopt the OCOM service mode during their routine work, and actual use refers to the frequency of doctors’ actual use. In this section, we describe the original UTAUT’s key factors that may play an important role in adopting the OCOM service mode.

#### 3.1.1. Performance Expectancy

According to the UTAUT theory, performance expectancy is defined as the degree to which an individual believes that utilizing a system will lead to improvements in their performance [19]. In this paper, we refer to performance expectancy as doctors perceive the benefits and advantages of using the OCOM service mode to provide online medical services. Some studies indicated that the performance expectancy positively impacts users’ intention to adopt and use technologies [36]. In the medical market, when doctors believe that the OCOM service mode can improve their performance, whether online or offline, they may be more receptive to and utilize this service mode. Given the discussion above, we argue that the relationship between performance expectancy and adoption intention is also true in the adoption of the OCOM service mode. Hence, we proposed the following hypothesis:

**H1.**  *Performance expectancy positively impacts doctors’ intention to use the OCOM service mode.*

#### 3.1.2. Effort Expectancy

The UTAUT model considers effort expectancy as a significant factor in the early stage of adoption, which indicates the ease level associated with using the system. Prior studies have shown that effort expectancy has a positive impact on users’ adoption intentions for new technologies [36]. In our study, the concept of effort expectancy refers to the degree to which a doctor believes using the OCOM service mode would require minimal effort. As users find it easier to operate a technology, they are more likely to perceive it as having higher “usefulness” [31]. Therefore, we assume that doctors are more likely to perceive the OCOM as a useful service mode for enhancing their performance when they can use it easily, which may increase their intention to use the OCOM service mode for online medical services. Hence, we proposed the following hypotheses:

**H2.**  *Effort expectancy positively impacts doctors’ performance expectancy for using the OCOM service mode.*

**H3.**  *Effort expectancy positively impacts doctors’ intention to use the OCOM service mode.*

#### 3.1.3. Social Influence

According to the UTAUT, social influence refers to the extent to which an individual believes that most people who are important to them think they should use the new technology or system [37], which is similar to the subjective norm in TAM2. Based on a study on technology adoption, social influence is a crucial factor in the adoption of new technologies [38]. Generally, social influence has a positive effect on the adoption intention to adopt new technology, such as mobile payment [39] and the health cloud [40]. In our study, social influence mainly refers to the support from doctors’ affiliated hospitals and internet platforms, which may impact doctors’ adoption of the OCOM service mode in their careers. Hence, we proposed the following hypothesis:

**H4.**  *Social influence has a positive impact on doctors’ intention to use the OCOM service mode.*

#### 3.1.4. Habit

According to an extended version of UTAUT, i.e., UTAUT2, habit is a critical factor influencing users’ intentions [41]. Habit refers to the process of learning that transforms into automatic responses triggered by specific cues, enabling effective achievement of particular goals [42]. Many studies have found that habits positively impact users’ adoption intentions toward new technologies [43]. In healthcare, doctors who frequently answer patients’ health-related questions or share health-related information and are active online may be more likely to adopt the OCOM service mode. Hence, we proposed the following hypothesis:

**H5.**  *Habit has a positive impact on doctors’ intention to use the OCOM service mode.*

### 3.2. Platform Experience and the OCOM Service Mode Adoption

Although a growing body of research exists on technology adoption and acceptance, most studies have been limited to examining the adoption behavior of a single product or service, which has failed to explain the relationship between multiple products or technologies. Ref. [21] claimed that the adoption of one technology depends on the presence of other related technologies, referring to such phenomena as “usage-transfer.” In the healthcare field, ref. [44] found that previously associated similar products can influence doctors’ initial impression and acceptance of smart healthcare. Before social platforms were involved in the medical field, doctors mainly provided medical services through professional online medical platforms. With the development of online medical services, social media platforms are increasingly popular due to their great potential in the medical field [15]. According to the theory of technology transfer, users generally estimate a new system based on their experience with the previous system, which impacts their intention to transfer to the new technology. In our context, the delivery of medical services via professional healthcare platforms and social media platforms may vary in terms of functionality and usage. Yet, the engagement of medical professionals in rendering online medical services or disseminating medical knowledge across these platforms can potentially influence and enhance one another. For example, doctors with experience providing online medical services on professional online medical platforms are more qualified and willing to provide medical services on social platforms, and vice versa. In this paper, different from the habit mentioned in Section 3.1.4, platform experience emphasizes the impact of usage experiences in some platforms on the willingness to use other platforms. Based on the discussion above, we proposed the following hypotheses:

**H6.**  *Platform experience positively impacts doctors’ intention to use the OCOM service mode.*

**H7.**  *Platform experience positively impacts the performance expectancy of the OCOM service mode.*

**H8.**  *Platform experience positively impacts the effort expectancy of the OCOM service mode.*

### 3.3. Future Expectancy and the OCOM Service Mode Adoption

Optimistic future expectancy is a positive outlook on technology, which has a positive impact on performance expectancy [45]. People who are optimistic about the use of technology are believed to have positive intentions regarding its use [46]. In our context, doctors may adopt the OCOM service mode to provide online medical services when they are optimistic about it. Optimism is typically considered a positive factor in the performance expectancy and effort expectancy of technology [47]. Doctors with an optimistic future expectancy are more likely to perceive the OCOM service mode as more useful and easier to use, as they worry less about possible negative future outcomes and expect more positive outcomes. Hence, we proposed the following hypotheses:

**H9.**  *Future expectancy positively impacts doctors’ intention to use the OCOM service mode.*

**H10.**  *Future expectancy positively impacts the performance expectancy of the OCOM service mode.*

**H11.**  *Future expectancy positively impacts the effort expectancy of the OCOM service mode.*

### 3.4. Patient Volume and the OCOM Service Mode Adoption

Medical services involve doctors and patients. According to indirect external network effects, an increase in the one-sided users’ adoption of the platform could impact the adoption intention of the other side users, such as sellers and buyers [48]. This phenomenon also exists in the healthcare field. For example, patient engagement positively impacts doctors’ online social capital return [8]. On an online platform, the extensive patient pool offers favorable conditions for doctors to generate profits and maintain their online business while providing expansive prospects for advancing their online medical careers. Based on the above discussion, increased patient volumes lead to the proliferation of online medical markets, presenting doctors with expanded opportunities to offer virtual medical services. Consequently, this trend amplifies doctors’ returns from online consultations. Therefore, it is reasonable to assume that doctors may select online platforms with large patient user volumes. Hence, we proposed the following hypothesis:

**H12.**  *Patient volume positively impacts doctors’ intention to use the OCOM service mode.*

### 3.5. Adoption Intention and the Adoption Behavior of the OCOM Service Mode

According to [26], individual behavior can be predicted through underlying intentions. Many studies have reported that adoption intention is a predictor and stimulus of behavior actions [49]. Hence, we further explore the usage behavior of the OCOM service mode, distinguishing between two types according to the platform: adoption of professional platforms and social platforms. Similarly, using intention is categorized by platform type to reflect the distinct channels. Hence, we propose the following hypotheses:

**H13a.**  *Adoption intention of professional platforms positively impacts doctors’ adoption behavior in this channel.*

**H13b.**  *Adoption intention to use social platforms positively impacts doctors’ adoption behavior in this channel.*

### 3.6. Interaction Effects Among Different Channels Use

After exploring the effect of platform experience on doctors’ intention to use the OCOM service mode in Section 3.2, we also focus on the interactive effects of actual use across different platforms. According to the principle of technology transfer theory, the experience of previous and similar technologies significantly influences users’ willingness to adopt and use the new technology [20]. In our context, doctors with experience in providing medical services on professional platforms may prefer to share health information and interact with other users, such as patients, on social platforms, and vice versa. It is consistent with the channel effect that activities on one platform may impact the use on other platforms. For example, releasing a short video on health science can increase doctors’ online consultation volume [17]. Hence, we propose the following hypotheses:

**H14a.**  *Doctors’ use of professional platforms positively impacts their use of social platforms for medical services.*

**H14b.**  *Doctors’ use of social platforms for medical services positively impacts their use of professional platforms.*

### 3.7. Moderating Analysis

**Figure 2:**
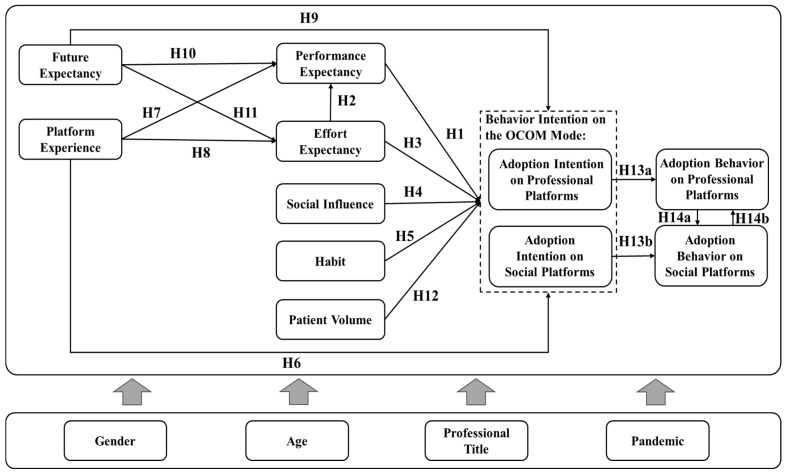
Research framework.

Users’ statistical characteristics are generally considered moderators of the relationships between the TAM and the UTAUT constructs, such as gender, age, income level, and education level [50]. In the healthcare setting, ref. [44] confirmed that the intentions of smart healthcare services vary between clinicians and non-clinicians. Ref. [51] explored the moderating effects of gender, age, professional title, and use experience in medical practitioners’ resistance and adoption of internet hospitals. In this paper, we argue that gender, age, and professional title may moderate the relationship between the key factors and doctors’ adoption intention of the OCOM service mode, which cannot be ignored in the context of online medical services. Particularly, the COVID-19 pandemic brought different conditions for doctors providing medical services compared to the pre-pandemic period, which may impact doctors’ adoption intention. Hence, we also focus on the moderating effects of the pandemic, which could provide valuable insights into the effective use of the OCOM service mode to deal with the surge in healthcare demand and the adoption framework for studying this technology. We use grouping models to test these moderating effects, as the factors in our study are represented by category variables. We visually depict the interconnections among the mentioned factors in Figure 2.

## 4. Research Methodology

**Figure 3:**
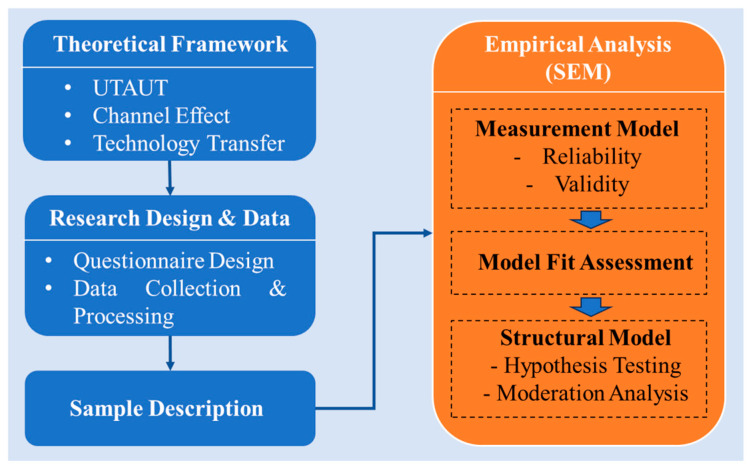
Methodological Procedure of this study.

This study follows a structured research procedure to examine doctors’ adoption of the OCOM service mode. Figure 3 presents the methodological procedure of this study, including questionnaire design, data collection, sample description, and SEM-based empirical analysis.

As shown in Figure 3, this study begins with the development of the theoretical framework, based on which the research hypotheses are proposed in Section 3. Subsequently, questionnaire design and data collection are conducted. The collected data are then processed and used to describe the sample characteristics. Finally, structural equation modeling (SEM) is employed for empirical analysis, including measurement model assessment, model fit evaluation, and structural model analysis. In this section, we first introduce the data collection process and the research sample, followed by descriptive statistical analysis to provide an overview of the dataset. Finally, we present the specification of the structural equation model, followed by the empirical analysis in Section 5.

### 4.1. Data Collection and Sample

We conduct a questionnaire survey, which includes two sections: (1) measuring respondents’ demographic information, followed by some questions related to the online medical service experience of online platforms for doctors, and (2) items measuring the construct proposed in Figure 2. In this questionnaire, the items are modified and adapted based on prior research to suit our study context. Specifically, items for doctors’ adoption intentions are adapted from Ref. [50,52]. Items for performance expectancy, effort expectancy, social influence, and habit for the OCOM service mode are adapted from the original scale created by Ref. [19,41]. Items for platform experience and future expectancy are adapted from Ref. [44,46]. These items are adjusted to match the context of the adoption of the OCOM service mode among doctors in our questionnaire. In addition, we develop two items to capture the degree to which doctors’ attention to patient volumes impacts their intention to use the platform for medical services. We measure these items using a five-point Likert scale, ranging from 1 (strongly disagree) to 5 (strongly agree). The survey was administered to physicians with experience in online medical services through WeChat. Data collection was conducted in May 2023. To ensure data quality, responses with missing values or logical inconsistencies were excluded. After data cleaning, a total of 958 valid responses were retained for the final analysis.

The theoretical framework of this study is grounded in the Unified Theory of Acceptance and Use of Technology (UTAUT), complemented by channel effect and technology transfer perspectives. In this study, we adopt key constructs from UTAUT, including performance expectancy, effort expectancy, and social influence, and extend the model by incorporating context-specific factors such as habit, platform experience, future expectancy, and patient volume. These constructs are operationalized into measurable variables and corresponding questionnaire items.

Table 1 reports adoption factors and their measurement items.

### 4.2. Data Analysis

**Figure 4:**
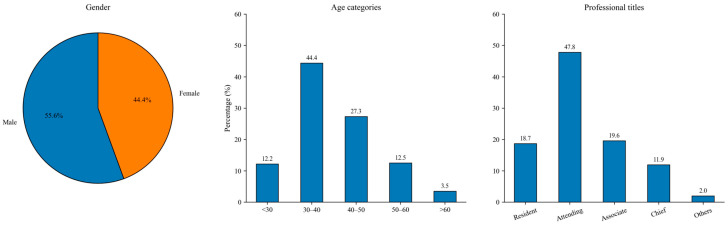
Distribution of respondents’ demographic characteristics.

The target survey object for this empirical research is a randomly selected doctor-group in China. We distributed questionnaires to these doctors and collected 958 valid responses, which allows us to observe people’s routine use intentions after the disaster without being affected by the strong shock of the pandemic. Data analysis is conducted in SPSS (v25). Table 2 shows the demographic information of the sample. To enhance readability and facilitate comparison, Figure 4 provides a visual representation of these distributions.

Structural equation modeling (SEM) is employed to examine the relationships among the constructs. Following the standard two-step approach, we first assess the measurement model and then evaluate the structural model. Detailed results of the analysis are reported in Section 5.

### 4.3. Model Specification

To enhance the formal rigor and clarity of the proposed framework, this section provides a mathematical specification of the structural equation model (SEM) employed in this study. Building upon the conceptual model and hypotheses developed in Section 3, we formalize the relationships among constructs using standard SEM notation.

#### 4.3.1. Measurement Model

In general, SEM consists of two components: a measurement model and a structural model. The measurement model describes the relationships between latent constructs and their observed indicators, which can be expressed as follows:

$$x_{ij}=\lambda_{ij}\xi_{j}+\delta_{ij}$$

$$y_{ik}=\lambda_{ik}\eta_{k}+\varepsilon_{ik}$$

where $x_{ij}$ and $y_{ik}$ denote observed indicators, $\xi_{j}$ represents exogenous latent variables, and $\eta_{k}$ represents endogenous latent variables. λ denotes factor loadings, while $\delta_{ij}$ and $\varepsilon_{ik}$ are measurement error terms.

Specifically, constructs such as effort expectancy (EEC), social influence (SI), habit (HAB), platform experience (PE), future expectancy (FE), and patient volume (PV) are modeled as exogenous latent variables. Constructs including performance expectancy (PEE), adoption intention toward professional platforms (AIP), adoption intention toward social platforms (AIS), and adoption behavior on professional and social platforms (ABP and ABS) are treated as endogenous latent variables. All constructs are operationalized using multi-item Likert-scale measures, as summarized in Table 1.

#### 4.3.2. Structural Model

Based on the hypotheses proposed in Section 3, the structural relationships among latent constructs can be expressed as a system of linear equations.

First, performance expectancy is influenced by effort expectancy, platform experience, and future expectancy:

$$PEE=\alpha_{1}EEC+\alpha_{2}PE+\alpha_{3}FE+\zeta_{1}$$

Second, adoption intention toward professional platforms (AIP) is determined by performance expectancy, effort expectancy, social influence, habit, platform experience, future expectancy, and patient volume:

$$AIP=\beta_{1}PEE+\beta_{2}EEC+\beta_{3}SI+\beta_{4}HAB+\beta_{5}PE+\beta_{6}FE+\beta_{7}PV+\zeta_{2}$$

Similarly, adoption intention toward social platforms (AIS) is specified as follows:

$$AIS=\gamma_{1}PEE+\gamma_{2}EEC+\gamma_{3}SI+\gamma_{4}HAB+\gamma_{5}PE+\gamma_{6}FE+\gamma_{7}PV+\zeta_{3}$$

Finally, adoption behavior on the two types of platforms is modeled to capture both intention effects and cross-channel interaction effects:

$$ABP=\theta_{1}AIP+\theta_{2}ABS+\zeta_{4}$$

(1) $$ABS=\phi_{1}AIS+\phi_{2}ABP+\zeta_{5}$$

where $\zeta_{1}$–$\zeta_{5}$ denote disturbance terms. These equations correspond directly to the hypothesized relationships (H1–H14) and the research framework illustrated in Figure 2.

#### 4.3.3. Estimation Strategy

The proposed model is estimated using covariance-based structural equation modeling (SEM) with maximum likelihood estimation. This approach allows for the simultaneous estimation of the measurement model and the structural relationships among latent constructs while accounting for measurement error.

Model estimation and hypothesis testing are conducted using IBM SPSS Amos (v28.0). Following the standard two-step procedure, the measurement model is first assessed in terms of reliability and validity, and the structural model is then evaluated to test the hypothesized relationships. Details are presented in Section 5.

## 5. Results

Structural equation modeling (SEM) was employed to examine the relationships among the constructs in the research model. Following the standard two-step approach, we first assessed the measurement model in terms of reliability and validity (see Section 5.1), and then evaluated the structural model to test the proposed hypotheses (see Section 5.2). Path coefficients and their significance levels were estimated to assess the relationships between constructs. This procedure ensures the robustness and transparency of the empirical analysis. We evaluate the structural model discussed utilizing Amos (v28.0), which offers a user-friendly graphical interface that facilitates the assessment of structural equation models. Details are shown below.

### 5.1. Measurement Model Assessment

To estimate the factors influencing doctors’ adoption of the OCOM service mode, we use Amos (v28.0) for the confirmatory factor analysis (CFA), testing the convergent, construct, and discriminant validity. Before testing the hypotheses, we analyze the measurement model, including reliability (Cronbach’s alphas and composite reliability) and validity (convergent and discriminant validity), to ensure the model’s rationality. We first analyze the descriptive information of respondents using SPSS (v25). We then report the means, Standard Deviation (SD), factor loadings, Cronbach’s alpha, composite reliability (CR), and Average Variance Extracted (AVE) values for the constructs in Table 3.

As shown in Table 3, the structure demonstrates internal consistency reliability, as indicated by Cronbach’s alpha, and the composite reliability of each construct exceeds 0.7, indicating good internal consistency. Furthermore, the factor loadings for each construct item were higher than 0.7, and the AVE values for all constructs exceeded 0.5, indicating acceptable convergent validity. Moreover, as shown in Table 4, all the constructs fulfill the Fornell–Larcker criterion, i.e., the square root of AVE for each construct is higher than its correlations with other constructs, showing the desired discriminant validity [53].

Following [54], we assess the model’s validity using maximum likelihood estimation. The Chi-Square/Degree of Freedom (ChiSq/df), Tucker–Lewis index (TLI), the goodness-of-fit index (GFI), the normed fit index (NFI), the comparative fit index (CFI), and the root mean square error of approximation (RSMEA) were used to evaluate the model fit. We report these fitness indices and acceptance criteria of the assessing model in our study in Table 5. We can find that all these indices are below or above the general threshold value, indicating that the model is a good fit for the data.

### 5.2. Structural Model Assessment

**Figure 5:**
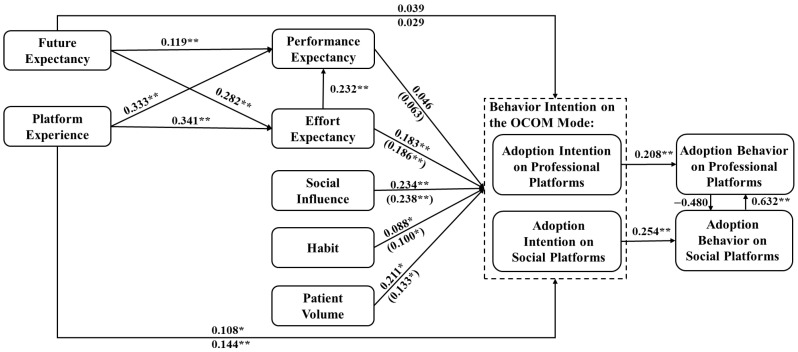
Structure model and path coefficients. Notes: Coefficients of paths to adoption intention on social platforms in parentheses. Significant at ** *p* ≤ 0.01, * *p* < 0.05.

We conduct a structural model evaluation to test the hypotheses using maximum likelihood estimation. The results are reported in Figure 5, including path coefficients and t-values. We can observe that performance expectancy does not affect doctors’ adoption intention of using the OCOM service mode, i.e., H1 is not supported. In our context, we focus on the intention of using the OCOM service mode in the post-pandemic era, and performance expectancy is no longer a critical factor that influences doctors to accept the OCOM service mode. Effort expectancy (H3: β = 0.183 (0.186), *p* < 0.001), social influence (H4: β = 0.234 (0.238), *p* < 0.001), and patient volume (H12: β = 0.211 (0.133), *p* < 0.01) have significant positive effects on adoption intention. Furthermore, effort expectancy has a significant effect on performance expectancy (H2: β = 0.232, *p* < 0.001), i.e., doctors will consider the OCOM service mode could enhance their performance if they perceive this model as user-friendly and requiring minimal effort to integrate into their workflow. Doctors’ internet habits also positively affect their intention (H5: β = 0.088, *p* < 0.05), i.e., doctors who frequently interact with patients online are more likely to accept the OCOM service mode. These results are consistent with the UTAUT model.

Interestingly, among these influence factors, social influence shows the strongest impact on adoption intention, suggesting that it is critical for the government and healthcare institutions to actively encourage doctors and patients to use the OCOM service mode. Robust social support from governmental entities, organizations, colleagues, and others can facilitate the embrace of innovative technologies, whether during a pandemic or in times of normalcy, which could be directed as an effort to popularize new technologies (Table 6).

Moreover, doctors with platform experience are more inclined to consider the OCOM service mode easy to use (H8: β = 0.341, *p* < 0.001), which can promote higher performance for doctors (H7: β = 0.333, *p* < 0.001). The experience of providing medical services through online platforms makes doctors more willing to accept the OCOM service mode (H6: β = 0.108 (0.144), *p* < 0.05) and obtain higher returns. Although doctors’ future expectancy of the OCOM service mode does not have a significant effect on the intention of using the OCOM service mode (i.e., H9 is not supported), it significantly impacts their performance expectancy (H10: β = 0.119, *p* < 0.001) and effort expectancy (H11: β = 0.282, *p* < 0.001) of adopting the OCOM service mode. This suggests that optimistic future expectancy may provide doctors with confidence, making it easier for them to use the mode. Hence, during the process of promoting this mode, focusing on its prospective development will incline doctors to be more receptive to comprehending and adopting this approach. Then, the intention positively impacts doctors’ behavior in the OCOM service mode, which includes professional and social platforms, supporting H13.

Finally, we emphasize the interactive effects of professional and social platforms on doctors’ use behavior. The results indicate that doctors’ use of social platforms for medical services can promote their use of professional platforms. Still, not vice versa, i.e., H14b is supported, and H14a is not. It can be seen that social platforms play a positive role in providing online medical care, which provides a new and convenient channel for doctors. This finding is consistent with the channel effect, where the use of social platforms can increase doctors’ consultation volumes on professional platforms [17]. More consultation volume means higher returns, which can attract more doctors [8]. Given this result, professional platforms and managers can encourage doctors to share health information on social platforms, which can enhance their performance and use on professional platforms. Moreover, social platforms may also function as exposure channels that increase physicians’ visibility and facilitate patient inflow, thereby indirectly promoting their engagement with professional platforms. This will significantly enhance the evolution of the OCOM service mode, thereby accelerating the progression of advanced online medical services.

### 5.3. Multi-Group Analysis

**Figure 6:**
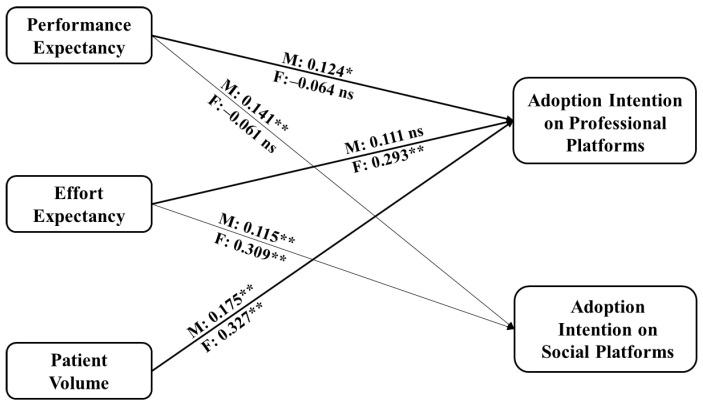
Path coefficient comparison between male and female doctors. Notes: ns means no significance; F refers to female doctor group results; M refers to male doctor group results; Significant at ** *p* ≤ 0.01, * *p* < 0.05.

We then examine the moderating role of some factors, i.e., gender, age, and professional title, in the doctors’ adoption of the OCOM service mode. Given that categorical variables indicate these moderators, we use grouping models to test their moderating effects. Figure 6 shows the moderating role of gender.

The results showed that doctors of different genders behave differently regarding the impact of performance expectancy and effort expectancy on doctors’ intention to use the OCOM service mode. In particular, female doctors’ performance expectancy has a negative but insignificant impact on their intention to use the OCOM service mode. This indicates that female doctors are less influenced by performance concerns when adopting the OCOM service mode. Still, compared to their male counterparts, they place greater emphasis on the user-friendliness of the service mode, that is, whether it is easy to use. Hence, to launch the medical service function on social or professional platforms, they first ensure ease of use. Female doctors also focus on the patient volume of professional platforms than male doctors when they choose to use a platform. Male doctors focus less on the platform’s ease of use and patient volume. These results provide insights into promoting a new service mode, specifically that the proportion of male and female users should be equal to avoid estimation bias caused by gender differences during the trial period of this new mode.

**Figure 7:**
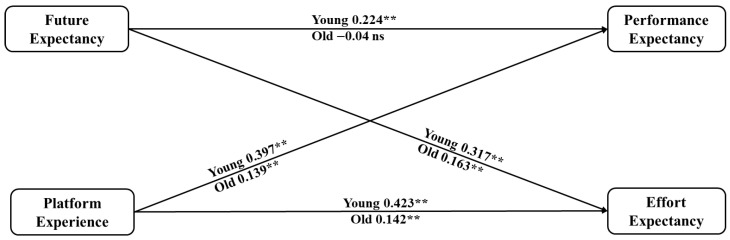
Path coefficient comparison between younger and older doctors. Notes: ns means no significance; Young refers to younger doctor group results; Old refers to older doctor group results; Significant at ** *p* ≤ 0.01.

Regarding doctors’ ages, we divided the doctors into a younger group and an older group, with 40 years old as the cutoff point, and then examined the differences between them. The results are reported in Figure 7, from which we can find that compared to older doctors, platform experience and future expectancy have stronger effects on younger doctors’ performance and effort expectancy. These results indicate that younger doctors focus more on the future development of the OCOM service mode, and their optimistic future expectations make them perceive the new service mode as more useful and easier to use. Hence, promoting the OCOM service mode for users of different ages should focus on varying perspectives. For younger doctors, for instance, it is essential to highlight the broad prospects of the OCOM service mode to address their concerns and boost their confidence in adopting the mode, thereby increasing their willingness to utilize it. Moreover, the initial promotion of selecting young doctors with experience in online medical platforms can yield better results.

**Figure 8:**
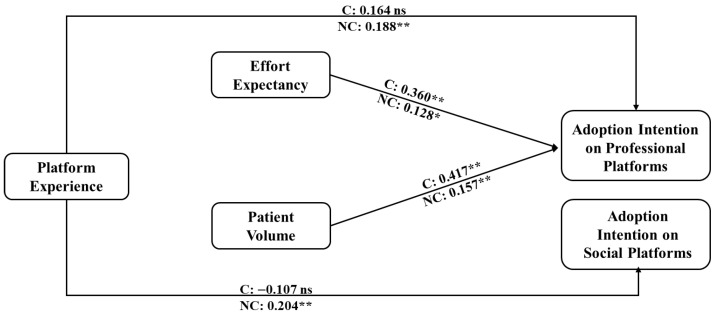
Path coefficient comparison between chief and non-chief doctors. Notes: ns means no significance; C refers to chief doctor group results; NC refers to non-chief doctor group results; Significant at ** *p* ≤ 0.01, * *p* < 0.05.

Moreover, we explore the difference between chief doctors (i.e., chief doctors and associate chief doctors) and non-chief doctors (i.e., resident doctors, attending doctors, and other doctors with lower titles) to examine the moderating effect of the professional title. As shown in Figure 8, chief doctors’ performance significantly affects their intention to use social platforms for medical services, but not professional platforms, while this factor does not affect non-chief doctors’ adoption intentions to use the OCOM service mode. Meanwhile, chief doctors focus more on the ease of using the service mode than other doctors. For non-chief doctors, platform experience influences their intention to use the OCOM service mode, which does not play a role in chief doctors’ use. Compared to non-chief doctors, the volume of patients on platforms affects chief doctors’ adoption intention more. Hence, to effectively encourage doctors to use the OCOM service mode, online platforms should prioritize user-friendliness and identify their target users by analyzing users’ habits and experiences. Additionally, increasing the volume of users on both the supply and demand sides is crucial for platforms to expand their market share.

### 5.4. Impact of the Pandemic

The COVID-19 pandemic, which occurred at the end of 2019, has changed the world, challenged the medical system, and brought some new technologies emerging because of the stay-at-home orders and health concerns. Ref. [18] found that situational constraints and health consciousness strongly positively affect the intention to adopt mHealth technology. The pandemic has promoted the development of online healthcare. In this context, doctors transfer their work manners, i.e., focusing more on online business than before the pandemic. In our paper, the pandemic effect refers to the pandemic’s positive or negative impact on the adoption of the OCOM service mode, which is distinct from the pandemic’s influence on the extent to which the mode is already in use. Therefore, we focus on the effect of the pandemic on the structure of doctors’ adoption of the OCOM service mode. Specifically, we divided respondents into two groups according to the period in which they mainly provided medical services online. If they provide medical services online always or never, they are classified as an unaffected group by the pandemic. Otherwise, they are considered affected by the pandemic.

**Figure 9:**
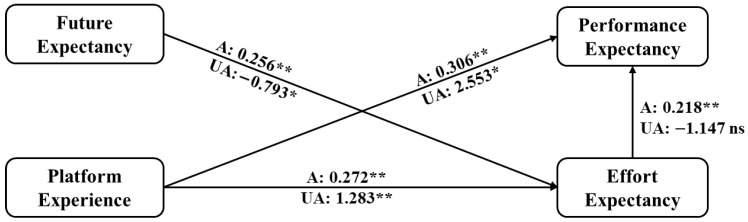
Path coefficient comparison between pandemic-affected and pandemic-unaffected doctors. Notes: ns means no significance; A refers to doctor results; UA refers to pandemic-unaffected doctor group results; Significant at ** *p* ≤ 0.01, * *p* < 0.05.

The results are reported in Figure 9. We can find that the pandemic moderates the path of effort expectancy to performance expectancy, indicating that the ease of using a new mode is critical for doctors affected by the pandemic. Meanwhile, doctors affected by the pandemic consider their platform experience and future expectancy may help them use the new mode more easily. Platform experience makes them believe in the usefulness of the mode more. During this period, doctors affected by the pandemic emphasize the support derived from the social environment, which alerts us and provides insights into how to utilize various online platforms for medical service delivery, i.e., reinforcing policy and workplace convenience for doctors.

## 6. Discussion and Implications

In this paper, from a technology adoption and use perspective, we aim to explore doctors’ adoption of the OCOM service mode for providing online medical services in the post-pandemic era. As shown in Figure 2, effort expectancy and social influence are significant predictors of adoption intention, while performance expectancy, traditionally emphasized in adoption models, shows no significant effect in the post-pandemic context. This suggests that doctors place greater emphasis on ease of use and demand-related factors when forming their adoption intentions, rather than relying solely on expected performance gains, implying that simplifying platform operations and making patient demand more visible may help translate these preferences into actual adoption behavior. In addition, platform experience plays a critical role, i.e., doctors’ prior engagement with professional or social platforms positively influences their intention to adopt the OCOM service mode. Notably, the use of social platforms reinforces the use of professional platforms, reflecting a channel effect [17] rather than substitution. This interaction confirms the relevance of technology transfer mechanisms [44], where familiarity with one system reduces perceived barriers to another, thus facilitating multi-platform adoption.

Second, this study explores the role of future expectancy and finds that, although it does not directly influence adoption intention, it has a significant indirect effect via effort expectancy. This highlights that while doctors may hold optimistic views of the industry’s future, their actual decisions are grounded in the immediate utility and usability of the system. Furthermore, moderation analysis reveals heterogeneity across demographic groups, such as gender, age, and professional title. These results provide a foundation for more nuanced platform strategies. For instance, platforms may consider differentiated engagement approaches by simplifying cross-platform operations and reducing the complexity of managing multiple service channels, particularly for doctors with limited digital experience. Providing structured guidance on content management and patient interaction across platforms can further lower the perceived effort required to use the system, thereby strengthening doctors’ intention to adopt the OCOM service mode. For doctors with senior titles, assigning professional online medical assistants may help reduce time costs associated with handling online consultations and platform maintenance, thereby improving their sustained engagement, particularly in high-demand periods. In addition, emerging AI-based assistance tools may further support doctors in managing routine tasks, such as patient triage, information organization, and content generation, which could reduce operational burden and enhance their intention to adopt the OCOM service mode. Beyond AI-based assistance, platform design features such as the integration of patient health records and automated information management may further streamline doctors’ workflows and reduce operational complexity, thereby enhancing perceived ease of use and supporting their engagement with the OCOM service mode.

Finally, this study offers implications for digital healthcare management in disaster contexts. The COVID-19 pandemic has reshaped medical service delivery by forcing doctors and patients to adopt new channels amidst travel restrictions and institutional strain. The results reveal that in such conditions, ease of use and social support mechanisms become particularly important, whereas long-term performance gains are deprioritized. These findings can inform the development of resilient digital health strategies during future crises. Policymakers and platform providers should thus prioritize reducing cognitive load, enhancing interoperability, and designing emergency-responsive service pathways to improve overall user experience. From the patient perspective, the integration of professional and social platforms may enhance accessibility to medical services, improve continuity of care, and support more flexible and timely interactions between patients and physicians. Overall, these findings highlight the broader value of omni-channel medical practice in improving healthcare accessibility and system resilience in increasingly complex service environments.

While this study provides important insights, several aspects may be further explored in future research. First, the use of self-reported survey data captures physicians’ perceptions of the OCOM service mode and may be subject to response bias; thus, future studies may complement these findings with behavioral or platform-based data. Second, the focus on physicians in China provides valuable context-specific insights, and extending the analysis to other healthcare systems could further enrich the understanding of cross-channel adoption. Third, the cross-sectional design reflects physicians’ adoption intentions at a particular point in time, and longitudinal research could provide deeper insights into the evolution of such behaviors.

## 7. Conclusions

This study investigates the behavioral mechanisms that influence doctors’ adoption of the omni-channel online medical (OCOM) service mode in the post-pandemic context. Grounded in the Unified Theory of Acceptance and Use of Technology (UTAUT), and informed by channel effect theory and technology transfer mechanisms, we develop an integrated framework that captures how platform-specific experiences and cross-channel interactions shape adoption intentions. The results demonstrate that effort expectancy, social influence, and usage habits remain central to doctors’ adoption decisions, while performance expectancy has become less influential under pandemic-driven digital shifts.

Our findings also reveal a directional spillover: doctors’ use of social platforms significantly promotes their engagement with professional platforms, but not vice versa. This asymmetry validates the channel effect in multi-platform healthcare environments and confirms the role of technology transfer in lowering cross-platform barriers. Additionally, our analysis of moderating factors shows that demographic characteristics and contextual exposure to the pandemic condition how doctors interpret and act on these behavioral drivers.

By articulating the distinct roles of cross-platform experience, contextual adaptation, and interaction effects, this study contributes a targeted explanation for OCOM adoption behavior. It provides a grounded theoretical basis for further exploring multi-channel healthcare engagement and offers a practical reference point for digital platform strategy in complex service ecosystems. In addition, the growing integration of AI-based assistance tools may further reshape doctors’ engagement in omni-channel medical services by reducing operational burden and enhancing perceived ease of use, providing a promising direction for future research.

## Figures and Tables

**Table 1:** Factors and measurement items.

Factors	Items
Performance Expectancy	Providing medical services on social (professional) platforms helps me gain more patient trust. Providing medical services on social (professional) platforms boosts recognition of my expertise among a wider audience. I believe providing medical services through social (professional) platforms benefits my career. Medical services on social (professional) platforms drive patients to consult me through professional (social) platforms.
Effort Expectancy	Using social (professional) platforms for medical services would be easy for me. I believe I can effectively manage and maintain both my social and professional platform accounts simultaneously. I believe patients recommended by social (professional) platforms fit my specialty.
Social Influence	I believe my company wants me to utilize social (professional) platforms for medical services. I think social (professional) platforms encourage me to offer medical services on them.
Habit	I regularly share medical content or answer questions on social (professional) platforms. I usually follow the social (professional) media accounts of some peers. Using social (professional) platforms for medical services is suitable for my internet habits.
Platform Experience	Experience with social (professional) platform medical accounts aids in managing professional (social) platform accounts.
Future Expectancy	I think more people will obtain medical services through social (professional) platforms. I think social (professional) platforms’ medical functions will gradually improve. I think more peers will offer medical services through social (professional) platforms in the future. I think the medical industry will focus more on social (professional) platforms.
Patient Volume	I prefer social (professional) platforms with more patient users over those with more peer users.
Adoption Intention of Social (Professional) Platform	I would continue using social (professional) platforms for medical services post-pandemic. I would use social (professional) platforms more frequently than before the pandemic.

**Table 2:** Demographic information of respondents.

Respondents’ Demography	Frequencies	Percentage
**Gender**		
Male	533	55.6
Female	425	44.4
**Age categories (years)**		
Young (below 30)	117	12.2
Early Middle-aged (30–40)	425	44.4
Middle-aged (40–50)	262	27.3
Late Middle-aged (50–60)	120	12.5
Old aged (above 60)	34	3.5
**Professional titles**		
Resident doctor	179	18.7
Attending doctor	458	47.8
Associate chief doctor	188	19.6
Chief doctor	114	11.9
Others	19	2.0

**Table 3:** Means, SDs, factor loadings, Cronbach’s Alphas, CR, and AVE of the constructs.

Constructs	Items	Mean	SD	Factor Loadings	Cronbach’s Alpha	CR	AVE
Adoption Intention to Professional Platform (AIP)	AIP1	3.78	1.18	0.78	0.77	0.76	0.61
	AIP2	3.78	1.13	0.78			
Adoption Intention to Social Platform (AIS)	AIS1	3.68	1.08	0.78	0.75	0.74	0.59
	AIS2	3.69	1.08	0.78			
Performance Expectancy (PEE)	PEE1	3.73	1.03	0.87	0.93	0.94	0.64
	PEE2	3.79	1.18	0.77			
	PEE3	3.66	1.10	0.79			
	PEE4	3.77	1.15	0.75			
	PEE5	3.69	1.14	0.81			
	PEE6	3.77	1.15	0.78			
	PEE7	3.68	1.11	0.81			
	PEE8	3.73	1.17	0.77			
Effort Expectancy (EEC)	EEC1	3.76	1.09	0.83	0.90	0.90	0.65
	EEC2	3.67	1.12	0.79			
	EEC3	3.80	1.16	0.76			
	EEC4	3.64	1.11	0.79			
	EEC5	3.79	1.14	0.77			
Future Expectancy (FE)	FE1	3.71	1.10	0.86	0.94	0.93	0.63
	FE2	3.74	1.17	0.79			
	FE3	3.66	1.11	0.79			
	FE4	3.80	1.18	0.76			
	FE5	3.71	1.15	0.80			
	FE6	3.77	1.19	0.79			
	FE7	3.70	1.11	0.80			
	FE8	3.74	1.18	0.79			
Patient Volume (PV)	PV1	3.69	1.04	0.83	0.81	0.82	0.69
	PV2	3.82	1.15	0.81			
Habit (HAB)	HAB1	3.74	1.08	0.84	0.92	0.92	0.65
	HAB2	3.83	1.19	0.77			
	HAB3	3.70	1.14	0.80			
	HAB4	3.82	1.17	0.75			
	HAB5	3.68	1.09	0.77			
	HAB6	3.82	1.19	0.77			
Social influence (SI)	SI1	3.72	1.05	0.84	0.88	0.89	0.66
	SI2	3.74	1.20	0.78			
	SI3	3.67	1.09	0.80			
	SI4	3.82	1.20	0.77			
Platform Experience (PE)	PE1	3.71	1.06	0.83	0.83	0.82	0.70
	PE2	3.80	1.18	0.85			

**Table 4:** Square roots of AVEs and collection matrix.

	AIP	AIS	PEE	EEC	SI	PE	FE	PV	HAB
Adoption Intention to Professional Platform (AIP)	**(0.78)**								
Adoption Intention to Social Platform (AIS)	0.39	**(0.77)**							
Performance Expectancy (PEE)	0.33	0.34	**(0.80)**						
Effort Expectancy (EEC)	0.41	0.41	0.43	**(0.81)**					
Social Influence (SI)	0.47	0.47	0.26	0.27	**(0.81)**				
Platform Experience (PE)	0.46	0.47	0.47	0.44	0.44	**(0.84)**			
Future Expectancy (FE)	0.39	0.37	0.32	0.40	0.42	0.34	**(0.79)**		
Patient Volume (PV)	0.46	0.41	0.26	0.27	0.36	0.45	0.41	**(0.83)**	
Habit (HAB)	0.40	0.40	0.25	0.26	0.42	0.41	0.43	0.46	**(0.81)**

Note: Square roots of AVEs in parentheses and bold.

**Table 5:** Fitness Index.

Fitness Indices	Threshold Value	Indices Values	Fitness Achievement
ChiSq/df	≤3.0	2.09	Achieved
TLI	≥0.9	0.96	Achieved
CFI	≥0.9	0.97	Achieved
NFI	≥0.8	0.94	Achieved
GFI	≥0.8	0.93	Achieved
RMSEA	≤0.08	0.03	Achieved

**Table 6:** Path coefficient estimation and hypotheses testing results.

Hypothesis: Path	Path Coefficient	T-Value	*p* Value	Supported? (Yes/No)
H1: PEE → AIP	0.046	1.126	0.26	No
PEE → AIS	0.063	1.514	0.13	No
H2: EEC → PEE	0.232	6.2261	<0.001	Yes
H3: EEC → AIP	0.183	4.299	<0.001	Yes
EEC → AIS	0.186	4.270	<0.001	Yes
H4: SI → AIP	0.234	5.419	<0.001	Yes
SI → AIS	0.238	5.403	<0.001	Yes
H5: HAB → AIP	0.088	2.057	0.040	Yes
HAB → AIS	0.100	2.264	0.024	Yes
H6: PE → AIP	0.108	2.206	0.027	Yes
PE → AIS	0.144	2.880	0.004	Yes
H7: PE → PEE	0.333	8.555	<0.001	Yes
H8: PE → EEC	0.341	8.829	<0.001	Yes
H9: FE → AIP	0.039	0.966	0.334	No
FE → AIS	0.029	0.699	0.484	No
H10: FE → PEE	0.119	3.514	<0.001	Yes
H11: FE → EEC	0.282	8.005	<0.001	Yes
H12: PV → AIP	0.211	4.424	<0.001	Yes
PV → AIS	0.133	2.760	0.006	Yes
H13a: AIP → ABP	0.208	4.892	<0.001	Yes
H13b: AIS → ABS	0.254	2.735	0.006	Yes
H14a: ABP → ABS	−0.480	−1.493	0.136	No
H14b: ABS → ABP	0.632	2.644	0.008	Yes

Notes: AIP, Adoption intention to professional platforms; AIS, Adoption Intention to social platforms; PEE, Performance expectancy; EEC, Effort expectancy; SI, Social influence; PE, Platform experience; FE, Future expectancy; PV, Patient volume; HAB, Habit; ABP, Adoption behavior to professional platforms; ABS, Adoption behavior to social platforms.

## Data Availability

The datasets used and/or analysed during the current study are available from the corresponding author due to privacy and ethical considerations.

## Abbreviations

OCOM	Omni-Channel Online Medical
UTAUT	Unified Theory of Acceptance and Use of Technology
TRA	Theory of Reasoned Action
TPB	Theory of Planned Behavior
TAM	Technology Acceptance Model
AIP	Adoption Intention to Professional Platform
AIS	Adoption Intention to Social Platform
PEE	Performance Expectancy
EEC	Effort Expectancy
SI	Social Influence
PE	Platform Experience
FE	Future Expectancy
PV	Patient Volume
HAB	Habit
